# Health care for patients with long COVID: a scoping review

**DOI:** 10.1590/1980-220X-REEUSP-2024-0056en

**Published:** 2024-10-25

**Authors:** Rafaela Deharo Curvelo, Ana Cristina Ribeiro, Sílvia Carla da Silva André Uehara

**Affiliations:** 1 Universidade Federal de São Carlos, Centro de Ciências Biológicas e da Saúde, Departamento de Enfermagem, São Carlos, SP, Brazil.

**Keywords:** COVID-19 Post Acute Syndrome, Evaluation of Results in Health Care, Patient Care, Health Services, Síndrome Post Agudo de COVID-19, Evaluación de Resultado en la Atención de Salud, Atención al Paciente, Servicios de Salud

## Abstract

**Objective:**

To map the scientific evidence on the care offered to health service users with Long Covid-19.

**Method:**

This is a scoping review based on the methods of the Joanna Briggs Institute. Primary studies were included, in Portuguese, English and Spanish, published between December 2019 and June 2023, in the Virtual Health Library, Web of Science, Scopus, PUBMED, SciELO and LITCovid LongCovid databases.

**Results:**

Of the ١٣ articles analyzed, it stands out that the care provided to patients with Long Covid is associated with drug prescription, indication of physical exercises, telerehabilitation and physiotherapy.

**Conclusion:**

A fragmentation was identified in the care provided to users of health services with Long Covid, with care directed only at isolated symptoms, without addressing the biopsychosocial care that people with this condition need.

## RESUMEN

**Objetivo:**

Mapear la evidencia científica sobre la atención ofrecida a los usuarios de los servicios de salud con COVID Larga.

**Método:**

Se trata de una revisión de alcance basada en los métodos del Instituto Joanna Briggs. Se incluyeron estudios primarios, en portugués, inglés y español, publicados entre diciembre de 2019 y junio de 2023, en las bases de datos Virtual Health Library, Web of Science, Scopus, PUBMED, SciELO y LITCOVID LongCovid.

**Resultados:**

De los 13 artículos analizados, destaca que la atención prestada a los pacientes con Covid larga está asociada a la prescripción de fármacos, indicación de ejercicios físicos, telerehabilitación y fisioterapia.

**Conclusiones:**

Se identificó una fragmentación en la atención prestada a los usuarios de los servicios de salud con Covid larga, con una atención dirigida sólo a los síntomas aislados, sin abordar la atención biopsicosocial que necesitan las personas con esta condición.

## INTRODUCTION

Once passed the acute phase of the SARS-CoV-2 virus infection, people affected by the disease began to report the manifestation of symptoms related to multisystem complications. These cases have been considered by the World Health Organization (WHO) as Long Covid, often occurring three months after the onset of Covid-19 while the symptoms may persist for at least two months and cannot be clarified by an alternative diagnosis^(1,2)^.

The symptoms of Long Covid-19 are highly heterogeneous and can compromise daily activities and work, as well as directly influencing the quality of life of those affected. Among the most common symptoms are persistent cough, fever, fatigue, dyspnea, chest pain, muscle pain, loss of smell or taste, changes in sleep patterns, cognitive impairment, anxiety and depression^(2)^

There are several hypotheses that explain the development of Long Covid-19, some of which are related to the existence of persistent reservoirs of SARS-CoV-2 in tissues, impacts on the microbiota and immune dysregulation^(3)^. However, due to its multifactorial condition and the diversity of symptoms, there is no standard protocol for the care of people affected by this condition, which makes the disease a challenge for health professionals, especially with regard to diagnosis and management^(1,4,5,6)^. Currently, a combination of treatments is offered, with pharmacological treatments aimed at relieving symptoms and non-pharmacological treatments covering rehabilitation to improve impaired functions, psychotherapeutic and social support^(3,7,8)^. In this context, the literature has described that, although the diagnosis of Long Covid-19 is usually made by a general practitioner, the management of these patients requires the cooperation of multi-professionals, approached in an individualized and biopsychosocial way, including the person affected by the disease in the self-management of health care^(8)^. Care for patients with Long Covid-19 requires a multi-professional approach focused on comprehensive care; however, as this is a new disease, it is not yet known how this care is being provided by health professionals, leaving a gap in knowledge. It is worth noting that in addition to compromising physical and mental health, Long Covid-19 can have a direct impact on daily activities, including work, as well as reducing quality of life and increasing demand on health services^(1,4–6)^. For these reasons, studies that investigate how health care is being provided to patients with Long Covid-19, whether it is within the principles of comprehensive, person-centered care or carried out in a fragmented, symptom-driven manner, are essential and innovative as they help to identify the potential and weaknesses of the current landscape of care for people with the disease, as well as serving as input for the strengthening and implementation of guidelines for the clinical management of these people. Therefore, this study aims to map out the scientific evidence on the health care offered to users of health services with Long Covid.

## METHOD

This is a scoping review based on the principles outlined by the *Joanna Briggs Institute* (JBI), which comprise the following stages: (1) formulation of the research question, (2) identification of relevant studies, (3) selection of studies, (4) data collection, (5) analysis, summary and reporting of results, and (6) dissemination of findings^(9)^.

### Definition of the Research Question

To conduct the search in the review, the JBI approach was adopted, represented by the acronym “PCC”, which covers the elements of “P” (population), “C” (concept) and “C” (context). The guiding research question was constructed based on the PCC strategy, with “P” (individuals with Long Covid), “C” (care and management) and “C” (health services), defined as follows: How is care for users with Long Covid being directed in health services?

### Search Strategy and Inclusion and Exclusion Criteria

The search for articles was carried out in the electronic databases: Virtual Health Library, *Institute for Scientific Information* (Web of Science), *Scopus*, *US National Library of Medicine National Institutes of Health*(PUBMED), SciELO and LITCovid LongCovid. The searches were carried out between February and March 2023, using descriptors and their synonyms from the Health Sciences Descriptor (DeCS) and *Medical Subject Headings* (MeSH), in different languages, namely: Long Covid, Nursing Care and Primary Health Care; and, in English, Rehabilitation, Long Covid and Exercise (Chart 1).

**Chart 1:** Search strategies used in the databases.

Database	Number of articles	Strategy
BIBLIOTECA VIRTUAL EM SAÚDE (VIRTUAL HEALTH LIBRARY)	59	(long covid) OR (post-covid conditions) AND (nursing care) AND (primary care) OR (primary healthcare) AND ( la:(“en” OR “pt” OR “es”)) AND (year_cluster:[2019 TO 2023])
	18	(covid de longo curso) OR (post-covid conditions) AND (nursing care) OR (cuidados de enfermería) OR (cuidados de enfermagem) AND (atenção primária à saúde) OR (primary health care) AND ( la:(“en” OR “pt” OR “es”)) AND (year_cluster:[2019 TO 2023])
WEB OF SCIENCE	643	post-acute sequelae of SARS-CoV-2 infection (Resumo) and nursing care (Resumo) and Primary Healthcare (Resumo) or Atención Básica (Resumo) or assistência de enfermagem (Resumo) or post-Covid conditions (Resumo) and Article (Tipo de documento) and English or Spanish or Portuguese (Idiomas)
	177	Long Haul Covid (Todos os campos) or post- Covid conditions (Todos os campos) and nursing care management (Todos os campos) and Cuidado Primário de Saúde (Todos os campos) or Atenção Primária à Saúde (Todos os campos) and Article (Tipo de documento) and English or Spanish or Portuguese (Idiomas)
SCOPUS	69	long AND covid OR post-covid AND conditions OR post-acute AND sequelae AND of AND sars- AND cov-2 AND infection AND nursing AND care AND management AND primary AND care OR primary AND healthcare AND ( LIMIT-TO ( PUBYEAR , 2023 ) OR LIMIT-TO ( PUBYEAR , 2022 ) OR LIMIT-TO ( PUBYEAR , 2021 ) OR LIMIT-TO ( PUBYEAR , 2020 ) ) AND ( LIMIT-TO ( DOCTYPE , “ar” ) ) AND ( LIMIT-TO ( LANGUAGE , “English” ) )
	13	long AND haul AND covid OR post-covid AND conditions AND nursing AND care AND primary AND care OR primary AND healthcare AND ( LIMIT-TO ( PUBYEAR , 2023 ) OR LIMIT-TO ( PUBYEAR , 2022 ) OR LIMIT-TO ( PUBYEAR , 2021 ) OR LIMIT-TO ( PUBYEAR , 2020 ) ) AND ( LIMIT-TO ( DOCTYPE , “ar” ) ) AND ( LIMIT-TO ( LANGUAGE , “English” ) ) AND ( LIMIT-TO ( SRCTYPE , “j” ) )
PUBMED	217	((“post acute covid 19 syndrome”[MeSH Terms] OR (“post acute”[All Fields] AND “covid 19”[All Fields] AND “syndrome”[All Fields]) OR “post acute covid 19 syndrome”[All Fields] OR (“long”[All Fields] AND “covid”[All Fields]) OR “long covid”[All Fields] OR (“post acute covid 19 syndrome”[MeSH Terms] OR (“post acute”[All Fields] AND “covid 19”[All Fields] AND “syndrome”[All Fields]) OR “post acute covid 19 syndrome”[All Fields] OR (“post”[All Fields] AND “covid”[All Fields] AND “conditions”[All Fields]) OR “post covid conditions”[All Fields]) OR (“post acute covid 19 syndrome”[MeSH Terms] OR (“post acute”[All Fields] AND “covid 19”[All Fields] AND “syndrome”[All Fields]) OR “post acute covid 19 syndrome”[All Fields] OR “post acute sequelae of sars cov 2 infection”[All Fields])) AND (“nursing care”[MeSH Terms] OR (“nursing”[All Fields] AND “care”[All Fields]) OR “nursing care”[All Fields] OR (“nursing”[All Fields] AND “care”[All Fields] AND “management”[All Fields]) OR “nursing care management”[All Fields]) AND (“primary health care”[MeSH Terms] OR (“primary”[All Fields] AND “health”[All Fields] AND “care”[All Fields]) OR “primary health care”[All Fields] OR (“primary”[All Fields] AND “healthcare”[All Fields]) OR “primary healthcare”[All Fields])) AND ((2019/12/1:2023/3/31[pdat]) AND (english[Filter] OR portuguese[Filter] OR spanish[Filter]))
	105	(((((“post acute covid 19 syndrome”[MeSH Terms] OR (“post acute”[All Fields] AND “covid 19”[All Fields] AND “syndrome”[All Fields]) OR “post acute covid 19 syndrome”[All Fields] OR (“long”[All Fields] AND “haul”[All Fields] AND “covid”[All Fields]) OR “long haul covid”[All Fields] OR (“post acute covid 19 syndrome”[MeSH Terms] OR (“post acute”[All Fields] AND “covid 19”[All Fields] AND “syndrome”[All Fields]) OR “post acute covid 19 syndrome”[All Fields] OR “post acute sequelae of sars cov 2 infection”[All Fields])) AND (“nursing”[MeSH Subheading] OR “nursing”[All Fields] OR (“nursing”[All Fields] AND “care”[All Fields]) OR “nursing care”[All Fields] OR “nursing care”[MeSH Terms] OR (“nursing”[All Fields] AND “care”[All Fields]))) OR (“atencion”[All Fields] AND (“drug effects”[MeSH Subheading] OR (“drug”[All Fields] AND “effects”[All Fields]) OR “drug effects”[All Fields] OR “de”[All Fields]) AND (“enfermeria”[Journal] OR “enfermeria”[All Fields]))) AND (“primary health care”[MeSH Terms] OR (“primary”[All Fields] AND “health”[All Fields] AND “care”[All Fields]) OR “primary health care”[All Fields] OR (“primary”[All Fields] AND “healthcare”[All Fields]) OR “primary healthcare”[All Fields])) OR ((“aten primaria”[Journal] OR (“atencion”[All Fields] AND “primaria”[All Fields]) OR “atencion primaria”[All Fields]) AND de salud[Investigator])) AND ((2019/12/1:2023/3/31[pdat]) AND (english[Filter] OR portuguese[Filter] OR spanish[Filter]))
	9	(“Rehabilitation”[All Fields] AND “long covid”[Title] AND “Exercise”[Title]) AND ((ffrft[Filter]) AND (fha[Filter]) AND (fft[Filter]) AND (english[Filter]))
SCIELO	4	(((long covid) OR (post-acute sequelae of sars- cov-2 infection) OR (post-covid conditions)) AND (nursing care)) AND (primary care) OR (primary healthcare) OR (primary health care) AND la:* AND type:(“research-article”)
	1	(post-covid conditions) OR (afecções pós-covid) OR (covid prolongado) OR (long covid) AND (assistência de enfermagem) OR (nursing care management) OR (cuidados de enfermería) AND (atenção primária à saúde) OR (primary health care) OR (primary healthcare) OR (atención básica) OR (asistencia sanitaria de primer nivel) AND la:* AND type:(“research-article”)
LITCovid LongCovid	330	(post-acute sequelae of SARS-CoV-2 infection) AND (nursing care management) AND (Primary Healthcare OR Primary Health Care) (LIMIT-TO TITLE) AND (LIMIT-TO ENGLISH) AND (LIMIT-TO PORTUGUESE) AND (LIMIT-TO SPANISH)

### Study Extraction and Selection

The primary studies included were in Portuguese, English and Spanish, published between December 2019 and June 2023, in the aforementioned databases. We excluded review studies, editorials, protocols, opinion articles, studies whose title and abstract did not answer the problem question, information from *websites* and media reports. The reference lists of all the studies found were also examined.

The references found were imported into the StArt (*State of the Art through Systematic Review*) application and selected on two levels: the first selection stage was analysis by reading the titles and abstracts, and then reading the full article. The StArt review tool was developed by the Software Engineering Research Laboratory (LaPES) at the Federal University of São Carlos^(10)^. Eligible studies were retrieved in full and assessed by three researchers. In both stages, differences were debated until a consensus was reached.

### Preparation of the Review

The preparation of this review also adhered to the PRISMA-ScR (*extension for scoping reviews)* recommendations for data extraction^(11)^. Relevant data was obtained from each article chosen, including authors, journal name, country where the study was carried out, country of publication, study design and main results. These results are presented in tables and in a descriptive format, addressing bibliometric aspects and answering the central question that guided this scoping review.

## RESULTS

**Figure 1:**
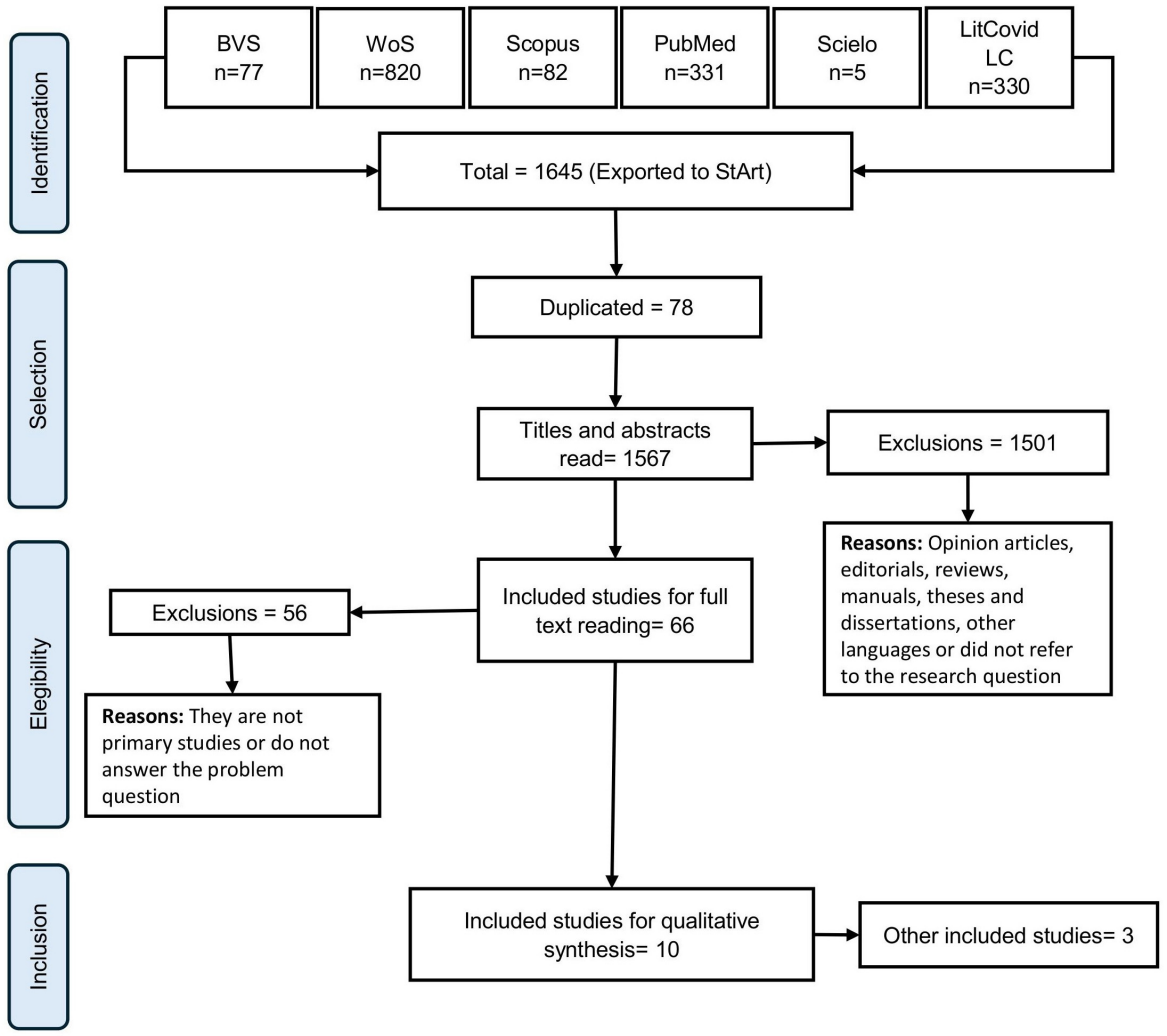
Reference flowchart: inclusion and exclusion of articles.

A total of 1,645 articles were identified from the databases. At this stage, there were 78 duplicate articles, and 1567 studies were selected for analysis of the title and abstract. From this analysis, 1501 studies were excluded because they were opinion pieces, editorials, reviews and manuals or did not answer the guiding research question. For the full reading, 66 articles were included and, finally, 10 studies were selected for the qualitative synthesis, while 3 articles were selected from the references of the selected studies (Figure 1).

A priori, studies on nursing care offered to patients with long COVID-19 in Primary Health Care (PHC) were selected, however, after a small number of studies addressing the topic were returned, the concept and context were expanded to general care and health services, respectively, and 3 articles were added, 1 of which was taken directly from the references of the selected studies and 2 from the US National Library of Medicine National Institutes of Health (PUBMED) platform.

The studies included in this review were published between December 1, 2019 and June 2, 2023, of which 4 (30.8%) were carried out in the United Kingdom and 1 (7.7%) in each of the following countries: United States, Chile, in several countries, Germany, Ukraine, Spain, Portugal, France and Austria; and the 13 (100%) articles were published in English.

Of the 13 studies selected, 3 (23.1%) were descriptive studies and randomized clinical trials, respectively (Chart 2).

**Chart 2:** Studies selected in the review, according to objective, type of study, main results and strategies and practices used by the studies to manage Long Covid.

Author	Objective	Type of Study	Main Results	Strategies and Practices Used
Dalbosco-Salas et al.^(12)^	To analyze whether the telerehabilitation program is effective in improving physical capacity, quality of life and symptoms in adult post-Covid patients.	Prospective observational study	Physical capacity, physical limitations, bodily pain, general health perceptions, vitality, social functioning, emotional limitations and mental health improved after the intervention in most patients. At the same time, there was a significant recovery in fatigue and dyspnea. Between individuals who had been admitted to the ICU and individuals who had not been admitted to the ICU, all aspects showed improvement, with the exception of bodily pain, general health perceptions, emotional limitations and mental health.	Telerehabilitation (offering physical exercises accompanied by professionals).
Daynes et al.^(13)^	To assess whether there are improvements in the variables fatigue, respiratory difficulty, exercise capacity and cognition after physical intervention, among individuals with Long Covid.	Observational study	The study indicated a decrease in exercise capacity and health-related quality of life compared to healthy controls, although anxiety, depression and cognition were preserved. The results indicate an improvement in fatigue, with an increase in the incremental and endurance walking test, Functional Assessment of Chronic Disease Treatment Fatigue Scale, EuroQual 5 domains (EQ5D) and Montreal Cognitive Assessment and a decrease in the Hospital Anxiety and Depression Scale	Continuous clinical monitoring. However, there is a lack of a specific rehabilitation protocol for patients with Long Covid-19.
Jimeno-Almazán et al.^(14)^	To compare the results of patients with Long Covid undergoing supervised therapeutic exercise intervention or following the WHO (World Health Organization) self-management rehabilitation leaflet.	Randomized clinical trial	A routine of simultaneous training sessions was carried out three days a week. Based on the prevalent symptoms (common to the condition, such as dyspnea and low-grade fever), the following variables were assessed: lung function, body composition (fat and lean body mass), quality of life and fatigue, anxiety and depression, cardiovascular health and muscle strength. After 8 weeks of physical exercise, there was a reduction in signs and symptoms, especially with regard to dyspnea. There was also a general improvement in all the variables; however, some of the results reported by the patients in the fatigue and quality of life variables did not show significant improvements compared to the control group, even though there was a partial improvement in dyspnea and lung functionality.	Physical fitness exercise interventions, with a focus on supervised and monitored physical rehabilitation.
Koliadenko et al.^(15)^	To study the clinical manifestations of psychopathological symptoms in Covid-19 survivors and develop a conceptual model to provide them with medical care in telemedicine.	Retrospective study	As far as mental health is concerned, none of the individuals studied had any indication of mental health or behavioral disorders prior to Covid-19. Around 98 patients (76% of all patients) had memory impairment. All the individuals assessed (with the exception of one) had considerable levels of anxiety, stress and depression at the time of the study, mainly as a result of the negative situation brought about by the Covid-19 pandemic. Hypochondriacal symptoms were also identified, resulting from low indicators of well-being, activity and mood. The treatment, based on the cognitive-behavioral approach and associated with the use of antidepressants and non-benzodiazepine tranquilizers, emphasized an improvement in levels of stress, anxiety and depression.	Cognitive Behavioral Therapy, with psychotherapeutic assistance provided through telemedicine, drug treatment and prolonged and continuous follow-up, with monitoring during and after the therapeutic intervention.
Nopp et al.^(16)^	To evaluate the efficacy and safety of outpatient pulmonary rehabilitation for patients who continue to have persistent or progressing respiratory and/or functional limitations after contracting Covid-19.	Prospective study	The change in the 6-minute walk distance increased (from the beginning to the end of rehabilitation). After 6 weeks of the rehabilitation program, the post-Covid-19 functional status scale decreased. Similarly, dyspnea measured with the mMRC scale decreased. In addition, patients improved in maximum workload and endurance capacity, and quality of life increased. Changes in lung function and respiratory muscle strength were explored after the initial data analysis: at the start of the study, the patients had impaired lung function in relation to age, gender and height-specific expected value, with a significant improvement by the end of the study. In addition, maximum inspiratory mouth pressure increased by 28%.	6-minute walk test, according to the guidelines of the *European Respiratory Society*.
O’Hare et al.^(17)^	To understand how physicians at the Department of Veterans Affairs (VA) handled the diagnosis of Long Covid and provided care to patients with suspected or confirmed Long Covid present in electronic health records (EHRs).	Cohort study	During the management of Long Covid, clinical uncertainty and fragmentation of care were observed. Clinical uncertainty refers to medical hesitation in diagnosing specific signs and symptoms of Long Covid. This is mainly due to the multiple etiologies of the post-Covid condition, the dynamics of the treatments received by patients, adverse health events that are not related to Covid-19, the lack of medical confidence in the patient’s clinical condition and the difficulty in monitoring patients affected by Long Covid. As for the fragmentation of care, the assistance offered to patients is isolated and poorly coordinated. This is due to the approach that gives little importance to comprehensiveness and patient concerns, the difficulty of multidisciplinary action and clinical recommendations that can be seen as undesirable or burdensome for patients.	The study does not mention the strategies commonly used, but highlights the practices that favor the fragmentation of care and the difficulties in diagnosing Long Covid.
Reis et al.^(18)^	To identify the aspects/components to be considered when planning and implementing telerehabilitation interventions that guarantee transitional care for people with Long Covid after hospitalization and to identify the positive aspects of telerehabilitation.	Descriptive study	In the study, the participating nurses brought up aspects of Long Covid-19 management that they considered relevant. Regarding coordination between the levels of care, the study participants emphasized the importance of maintaining continuity of care, but with transitional care, maintaining empathetic listening in order to identify the health needs of the community and help individuals participate in their health-disease process. Telerehabilitation favors this transitional care, as it is still possible to ensure all interventions in an e-health modality, however, face-to-face moments are pertinent to evaluate respiratory and motor rehabilitation programs, which should be carried out with exercises that increase ventilatory capacity, chest expansion, diaphragm performance, control of associated symptoms (cough, dyspnea and expectoration) and tolerance to exertion. Motor rehabilitation, on the other hand, should encourage muscle strength, flexibility, joint range of motion, improved walking ability and quality. In addition, telerehabilitation has proved to be important in terms of moving individuals around, since it is possible to manage health conditions in one’s own home, with the help of the health team.	Coordination of integration between Specialized Care and Primary Care. Addressing multidisciplinarity and telerehabilitation.
Romanet et al.^(19)^	To evaluate the effectiveness of physical training on dyspnea and health-related quality of life in individuals with Long Covid.	Randomized controlled trial	The average Multidimensional Dyspnea Profile (MDP) score after physical training rehabilitation was 42% lower than after standard physical therapy. In addition, significant reductions were observed in the MDP subcategories: respiratory discomfort, sensory dimension and emotional response. Thus, the results suggest that physical training rehabilitation had positive effects on dyspnea and quality of life compared to standard physiotherapy in patients previously hospitalized with acute respiratory distress syndrome due to Covid-19.	Specific physical training (walking and resistance and muscle strength training) and standard physiotherapy.
Schrimpf et al.^(20)^	Evaluate the current number of patients with Long Covid (patients with symptoms between 4 and 12 weeks and more than 12 weeks) treated by general practitioners, as well as the symptoms most frequently observed in patients with acute Covid-19 and Long Covid by these physicians.	Descriptive study	Regarding the management of Long Covid-19, 97.2% of the health professionals interviewed had already treated a case of persistent symptoms between 4 and 12 weeks of Covid-19. The ability to diagnose these patients was determined to be 62.8%, while therapeutic options are rated at around 47.4%. Around 79.6% of professionals claim to have had contact with patients who have had symptoms for more than 12 weeks, with diagnostic capacity at 40.7% and therapeutic options at 54.9%. Professionals reported that around 18.3% of all Covid-19 patients need a certificate because they are unable to work, and approximately 3.7% of patients have access to rehabilitation centers. Among the treatments discussed, there are drug options, non-drug options (such as physiotherapy) and/or referrals to specialized services.	Drug and non-drug therapies, physical rehabilitation and referrals to specialized services.
Nurek et al.^(21)^	To provide a quick expert guide to doctors and clinical services in Long Covid, starting with the development of a list of recommendations.	Descriptive study	Among the results, we highlight the importance of clinical knowledge about the etiology, making the diagnosis based on pre-established criteria, individualized investigations, considering that each individual has their own health needs, appropriate referrals to specialty centers, complete evaluations, with specific tests for each case and rehabilitation activities for the management of signs and symptoms, which include educating the patient about their health condition, encouraging them to participate in their own health-disease process and make use of therapeutic options (alternative or not), such as physiotherapy, drugs, etc.	Multiprofessional and interprofessional approach, referrals to specialized services, considering the physiological, psychological and social aspects of recovery.
Ladds et al.^(22)^	To develop a model for the management of Long Covid, based on the experiences of health professionals.	Qualitative study in which participants were asked to choose between an individual narrative interview or participation in an online focus group	In this study, we developed a suggested model for improving the character of care at Long Covid, which addresses referral criteria, referral from the family doctor or hospitalization team, telephone screening, with investigations that consider systemic factors, guaranteeing integrality, and offer care from different medical specialties and the development of monitoring, follow-up and cognitive stimuli.	Multidisciplinarity, with continuing education practices and integration between services and continuity of care, with therapeutic and clinical follow-up.
Jimeno-Almazán et al.^(23)^	To determine the effectiveness of physical exercise, respiratory muscle training and the World Health Organization (WHO) recommendations leaflet on the recovery of physical fitness, quality of life and symptom status in people with post-Covid-19 syndrome.	Cohort study with randomized clinical trial	After 8 weeks of intervention, which consisted of a supervised concurrent training program (with or without inspiratory muscle training), there was a significant improvement among all the participants in the study. There was a decrease in individuals who reported having moderate and severe symptoms, especially among individuals who performed physical exercise (compared to those who only performed breathing training and the controls). There was a low decrease in the control group (except for the dyspnea factor, which increased), but there was a considerable improvement in symptoms in the group that participated in physical exercise and a median improvement in the groups that performed respiratory muscle training (associated or not with physical exercise).	Practicing physical exercise.
Humphreys et al.^(24)^	Explore the lived experience of Long Covid, focusing on the role of physical activity.	Qualitative study using semi-structured interviews	For some participants, prolonged physical disability had an emotional impact on them, causing low self-esteem, frustration and guilt at not being able to carry out daily activities. Participants had varying expectations of how the health system could assist them with physical activities, with many feeling unassisted by their medical professional and seeking information online. In addition, as physical or cognitive activities resulted in the onset of fatigue, participants reported a loss of freedom to engage in everyday activities. Thus, when performing physical exercise, these individuals reported relapses, which held a small perceived improvement in baseline function, because it was considered a price worth paying for the sense of normality, control and positive effect the activity provided, for fear of adverse effects or on medical advice. Most participants established personal strategies for managing physical activity and many expressed a desire for better monitoring and support to manage physical activity. Most were unable to resume activities that were once central to their core identity, so any activity that provides a sense of normality helps to refute the idea that this altered identity is permanent.	Adoption of physical activity with individualized care and continuous monitoring, as well as emotional support to help with the impact of daily functions.

The articles analyzed in this study looked at the clinical management of patients with long COVID-19, with 10 (77%) studies looking at the effectiveness of physical exercise and physiotherapy in improving signs and symptoms.

The studies highlighted the adoption by health services of a one-off care approach, focused on the signs and symptoms of long COVID-19 and with a biomedical approach. In this context, 6 (46.2%) studies assessed clinical knowledge for the management of post-Covid conditions; and only 3 (23.1%) addressed referral to specialized services.

Telerehabilitation, which was frequently discussed, was a strategy adopted by health services for the management and monitoring of biopsychosocial conditions associated with the long Covid condition, by 3 (23.1%) studies. The focus on psychological demands was better addressed in 1 (7.7%) study, although these demands are mentioned in other studies, which report a considerable improvement in this variable after the proposed interventions. In addition, the selected studies did not provide specific guidelines for managing patients with long COVID-19. They only analyzed actions aimed at a limited field of action and did not focus on analyzing care for signs and symptoms in a more comprehensive way.

## DISCUSSION

The studies examined in this review stress the efforts to identify health care actions aimed at understanding the etiological and health factors of Long Covid, considering the clinical evaluation and forms of treatment offered to patients.

The persistent symptoms of Covid-19 presented most frequently among the studies evaluated are respiratory disorders, such as dyspnea, difficulty breathing and coughing; as well as memory impairment, less ability to deal with frustrations, anxiety, depression, panic attacks, obsessive-compulsive disorder and mental confusion. In addition, many individuals suffer from fatigue, weakness, myalgia, reduced physical capacity, dysgeusia and anosmia^(12–20)^.

However, the conduct and management of Long Covid-19 are still considered imprecise, since there is clinical uncertainty, especially regarding the identification of these cases, which is related to the hesitation of health professionals in diagnosing specific signs and symptoms associated with the clinical condition. This has an impact on medical conduct, mainly due to the multiple etiologies underlying the post-Covid condition, the variety of treatments offered to health service users, the adverse health effects that are not related to Covid-19, the lack of medical confidence in the patient’s clinical condition and the difficulties in monitoring individuals affected by Long Covid^(17)^.

Thus, since the investigation of possible alternative diagnoses to Long Covid-19 is limited in health services, health professionals should consider the occurrence of multisystemic disorders, and it is pertinent to investigate possible causes underlying the symptoms of this clinical condition^(21,22,23)^. Therefore, it is important to carry out an initial screening, paying attention to the various signs and symptoms and considering the possibility of diagnosing Long Covid in patients who have had clinical confirmation of Covid-19 or a history of a positive test with new or evolving symptoms^(13,21)^.

It is noteworthy that individuals with Long Covid without a previous laboratory diagnosis of Covid-19 represent a challenge in clinical practice, since many affected by SARS-CoV-2 are asymptomatic or have not undergone RT-PCR testing, which makes the diagnosis of Long Covid more difficult to make. In addition, the variety of symptoms also interferes with the diagnosis of Long Covid-19, since patients can present with anything from the most common symptoms, such as fatigue, difficulty breathing, coughing, joint discomfort, chest pain, muscle pain and headaches, to other rarer symptoms^(25)^.

Nevertheless, due to the prolonged uncertainty about the cause of the symptoms, the progression and the clinical treatment of Long Covid symptoms, some healthcare professionals adopt additional testing and referral to specialists in order to obtain a more detailed assessment. Such approaches can include imaging or diagnostic tests to monitor the service user’s recovery and explore possible alternative causes for the symptomatology, as well as follow-up in specialized services^(17,21,22)^.

Similarly, in order to optimize care for Long Covid-19, it is essential to value clinical knowledge of the underlying cause. In this context, it is worth noting that many service users feel that the advice provided by health professionals is insufficient or even does not fit in with what they want to hear and they look for information on the internet, not always on pages with reliable content, which has consequences for their health. As a result, training health professionals is essential, especially with regard to the identification and clinical management of Long Covid-19, in order to provide comprehensive and effective care^(22)^.

Therefore, the health services analyzed by this study refer to the importance of diagnosis based on scientific and defined criteria, “personalized” clinical assessments for each user, appropriate referrals to specialists, complete investigation through specific tests, education and encouragement of the person to actively participate in managing their own health, and of the health professional to be able to offer reliable, practical and easy-to-understand guidance^(21)^.

Thus, the way to diagnose Long Covid-19 depends on the symptoms presented by the individual. Thus, in the case of pulmonary manifestations, clinical assessment, pulmonary function tests and chest X-rays should be followed, with the recommendation of a CT scan in cases of X-ray alterations or clinical impairment of the lungs. In addition, it is necessary to examine the service user and carry out non-invasive tests (CT, echocardiography or MRI) for cardiac complications, blood counts for vascular complications, blood and urine tests and, in abdominal cases, consider the need for CT or MRI. In neuropsychiatric conditions, the investigation is carried out by screening for signs of stress, anxiety and depression and, if necessary, by CT scan, electroencephalogram or MRI^(26)^.

Despite this, referrals to these specialized services face coordination and communication challenges between health care levels, especially in cases of more complex symptoms^(18,20)^. The care offered by health services is often associated with the therapies previously used by users, without integration with other aspects of treatment. In addition, there is an unintelligible delineation of roles between the multidisciplinary team and specialist doctors, which hinders effective collaboration between them^(17)^.

Thus, the analysis of the studies selected in this review reveals the fragmentation of care related to the clinical management of Long Covid-19, in which the care offered to users is carried out in isolation and with inadequate coordination between the health services. This is due to care approaches that are not concerned with comprehensive health care and the individual afflictions of patients, let alone the difficulty of implementing multidisciplinary approaches and clinical guidelines that may be undesirable or costly for them^(17)^.

Against this backdrop, a study carried out in PHC services in Portugal showed that nurses sought to ensure continuity of care for users with Long Covid-19 by coordinating levels of care (Hospital Care and PHC), including visits before hospital discharge to assess contexts related to the health-disease process, as well as to prepare the family for discharge, in order to avoid defragmentation of care and interruptions in the rehabilitation program^(18)^.

However, under the Brazilian Unified Health System (SUS), both continuity of care and referral to specialized services face several challenges, such as delays in secondary services and long waiting times for referral appointments, which cause inefficiency in clinical care and worsen the clinical situation of users of these health services. In addition, the low frequency of home visits to disabled patients, the lack of a solid doctor-patient relationship and poor communication between health professionals have a negative impact on the quality of care provided to PHC users. There are also biopsychosocial factors, which include disabling conditions that make travel difficult, pain that makes it hard to get around, dependence on family members for access to appointments, difficulty accessing public transport, low family income and lack of financial and logistical support from the family^(27)^.

In this context, telerehabilitation has been highlighted as a key alternative to dealing with health needs arisen since the onset of Long Covid-19. Thus, remote rehabilitation has shown its relevance to the mobility and displacement of individuals, allowing them to manage their health conditions from the comfort of their homes and with the support of the healthcare team. Thus, for people who face mobility difficulties, accessibility to Specialized Care can be difficult to manage and telerehabilitation emerges as a solution, using communication technologies to offer continuous support and rehabilitation to people in geographically distant areas. It should be noted that this method is not intended to replace traditional assessments, but to provide an alternative approach to health care, and was already used in the pre-pandemic period, but was widely disseminated during and after the critical phase of Covid-19^(12,18,22,28)^.

In terms of advantages, home-based telerehabilitation is an option adopted by health services when used to monitor or assess users of these services during corrective therapy, which is beneficial for individuals with serious conditions, providing physiotherapy at home and avoiding travel to a health unit. Despite this, inequality in access to this technology is a significant problem, especially in low- and middle-income countries, and there can also be a lack of direct human contact with the doctor, since clinical interaction is carried out through technology. In addition, for each user, health professionals need to adapt the remote intervention to meet individual treatment needs^(29,30)^.

Prior to the Covid-19 pandemic, a study carried out in the United States with wheelchair users showed that a telerehabilitation program mitigated obstacles associated with conventional care, such as travel from their own homes and access to specialists. Through interactive sessions and real-time assessments, the program offered effective self-management education. In addition, observing participants in their home environments helped identify and manage likely obstacles that could potentially affect their treatment, resulting in faster approaches^(31)^.

Likewise, telerehabilitation approaches are just as effective as face-to-face ones, especially in programs for managing chronic conditions^(32)^. Telerehabilitation favors transitional care, since it is possible to maintain care interventions in an *e-health* modality, although there are times when face-to-face interaction is necessary, especially to assess and carry out respiratory and motor rehabilitation programs^(18)^.

In this way, telerehabilitation improves access to rehabilitation programs, offering greater comfort to patients with Long Covid-19 and providing continuity of care. It should be noted that the critical phase of the pandemic has boosted the adoption of remote health solutions, allowing health professionals to adapt to the new reality and offer more comprehensive and accessible support to health service users, including rehabilitation programs according to individual needs. Furthermore, in the context of Covid-19, telerehabilitation encourages greater autonomy and control over one’s own health-disease process, including self-management strategies. From this perspective, the inclusion of a telerehabilitation program, including self-management by health services, can provide an improvement in physical capacity, general health, vitality, social function and mental health in some patients affected by Long Covid-19, as well as fatigue and dyspnea^(12,18)^.

On the other hand, one study emphasized the need for face-to-face care for people with Long Covid-19, due to the need for continuous monitoring, comprehensive care and analysis of signs and symptoms, as well as the difficulty in carrying out clinical assessment and communication, caused by the remote modality^(22)^.

At the same time, moderate physical exercise rehabilitation is often indicated, with the aim of relieving symptoms and improving physical performance (although it is necessary to consider the limitations and possible recurrence of clinical manifestations of Long Covid-19, requiring individualized action plans in order to avoid the return of signs and symptoms). In this sense, a multidisciplinary rehabilitation approach is ideal, covering a holistic perspective that encompasses the complex sequelae of SARS-CoV-2 infection^(20)^.

Studies on rehabilitation from Covid-19 through physical exercise have also shown a reduction in negative feelings such as anxiety and depression, significant improvements in quality of life, a return to daily activities and a reduction in symptoms such as coughing, fatigue, memory impairment and dyspnea. It is noteworthy that high-intensity exercise (i.e. longer sessions with higher intensity target exercises) is considered the most appropriate type of rehabilitation treatment for individuals with shortness of breath after Covid-19, since quality of life related to physiological health improves when associated with this variable^(13,19)^.

In an 8-week intervention in an outpatient service, there was a reduction in the number of people reporting moderate and severe symptoms of Long Covid, especially among those who practiced physical exercise^(23)^. In addition, the functional conditions of post-Covid patients in the physical rehabilitation service improved, with a reduction in prevalent symptoms of the condition such as dyspnea, fatigue, anxiety and depression, an increase in endurance capacity, body composition and quality of life, and there was also a significant recovery in lung function and respiratory muscle strength^(14,16)^.

Fatigue resulting from physical or cognitive activities can limit participation in everyday tasks, leading to a feeling of loss of freedom. Thus, despite relapses with physical exercise, most people consider the physical effort to be compensatory, due to the sense of normality and control, seeking activities to counterbalance the adverse effects^(24)^.

In this context, associated with physical rehabilitation, there is the adoption of medication as a management strategy for Long Covid-19, such as non-opioid analgesics (paracetamol and ibuprofen) to relieve headaches, as well as the use of corticosteroids or bronchodilators to treat some respiratory symptoms^(20)^. There are also considerations about the use of antidepressants to relieve persistent inflammatory symptoms, with a focus on reducing inflammatory markers and restoring immune function^(33)^. Furthermore, in order to reduce the persistence of phlogistic signs and symptoms, patients with Interstitial Lung Disease (ILD) caused by Long Covid have been using prednisone, which leads to a rapid and significant improvement in shortness of breath and lung function^(34)^.

Vitamin supplements are also recommended, especially vitamin D and B vitamins^(20)^. Therefore, it is essential to carry out a complete evaluation through anamnesis, medical history and tests, in order to obtain a comprehensive assessment, including blood tests, kidney function, C-reactive protein, liver function, thyroid, hemoglobin A1c (HbA1c), vitamins D and B12, magnesium, folate and ferritin levels^(33)^.

In the Brazilian context, the importance of PHC with regard to the management of Long Covid-19 is evident, since individuals with persistent Covid-19 symptoms tend to seek care at this level of care for clinical evaluation, tests and, if necessary, referral for rehabilitation by other specialties, as well as referral to Specialized or Hospital Care^(35)^.

Despite this, a minority of patients are able to access health rehabilitation services. It is therefore necessary to guarantee access to intervention actions, in line with the principles of the SUS, and also to maintain continuity of care. Another challenge is the lack of knowledge about Covid-19, which can lead to poor adherence to rehabilitation services, since many service users may neglect their symptoms. Furthermore, there is evidence of a lack of preparation and organization of Brazilian health services during the health emergency phase imposed by Covid-19, as well as a lack of knowledge and standardization of care for people who present symptoms characteristic of Long Covid^(36)^.

In addition, it is necessary to maintain interdisciplinary care, with the aim of carrying out not only physical rehabilitation, but also mental and social rehabilitation^(37)^. The psychological rehabilitation of persistent Covid-19 symptoms in the health services that adopt it refers to a treatment based on the ­cognitive-behavioral approach and complemented by non-benzodiazepine antidepressants and tranquilizers, which emphasizes an improvement in stress, anxiety and depression levels, although in situations of cardiovascular, excretory complications or other systemic manifestations during the acute phase of Covid-19, greater cognitive complexity occurs, requiring deeper psychological support^(15)^. Thus, the biopsychosocial perspective of health is the most effective strategy for providing comprehensive care, taking into account the psychological and social factors of the service user, not just the biomedical aspects^(38)^.

Despite this, physical exercise, especially aerobic exercise, adopted as a strategy by health services, shows substantial improvements in the mental health of individuals with Long Covid, compared to those undergoing traditional treatments or who do not practice activities. This is because exercise can trigger a series of interconnected changes in the brain, resulting in an environment that offers protection against depression and anxiety^(39)^.

Therefore, it is essential to ensure access to services that promote mental health for patients with Long Covid, and it is necessary to identify those who require additional support and refer them to specialized treatment. However, it is important to avoid pathologizing the individual, considering that the physical symptoms of Covid-19 can influence the assessment^(40)^. Thus, although there are protocols for isolated strategies, such as physical rehabilitation, it is necessary to develop and implement specific management guidelines that take into account the comprehensive health care of patients affected by Long Covid^(13)^.

In this study, the results show the long-term reverberation of the Covid-19 pandemic and the impact on health services, requiring the formulation of guidelines related to the care and treatment of Long Covid, in addition to the need to direct resources and efforts towards more effective approaches, since there is a clear gap in the literature on how to identify and clinically manage people with this condition.

## CONCLUSION

This scoping review allowed us to identify that care for patients with Long Covid-19 in health services has been significantly focused on the signs and symptoms of the disease, permeated by the biomedical model, thus highlighting the absence of the comprehensive care that the person who develops the disease needs, given that the impacts of the disease go far beyond physical health. Therefore, this study contributes to strengthening the theoretical framework on the fragility of comprehensive care for patients with Long Covid-19 in health services, as well as to the development and implementation of protocols for the clinical management of the disease that guide the importance of multi-professional coordination in the comprehensive care of these people. Finally, it is suggested that future studies work on identifying the difficulties faced by health professionals in providing comprehensive care to health service users with Long Covid-19.
