# Supplementary material for: Health care for patients with long COVID: a scoping review
Source: Rev Esc Enferm USP. 2024 Oct 25;58:e20240056. doi: 10.1590/1980-220X-REEUSP-2024-0056en (PMC11524158; doi:10.1590/1980-220X-REEUSP-2024-0056en)
Supplement: Translation

# Translation: Assistência à saúde de pacientes com COVID longa: uma revisão de escopo

**DOI:** 10.1590/1980-220X-REEUSP-2024-0056pt

**Authors:** Rafaela Deharo Curvelo, Ana Cristina Ribeiro, Sílvia Carla da Silva André Uehara

**Affiliations:** 1 Universidade Federal de São Carlos, Centro de Ciências Biológicas e da Saúde, Departamento de Enfermagem, São Carlos, SP, Brasil.

**Keywords:** Síndrome de COVID-19 Pós-Aguda, Avaliação de Resultados em Cuidados de Saúde, Assistência ao Paciente, Serviços de Saúde

## Abstract

**Objetivo:**

Mapear as evidências científicas sobre a assistência ofertada aos usuários dos serviços de saúde com Covid longa.

**Método:**

Trata-se de uma revisão de escopo com base nos métodos do Joanna Briggs Institute. Foram incluídos estudos primários, nos idiomas português, inglês e espanhol, publicados no período de dezembro de 2019 a junho de 2023, nas bases de dados Biblioteca Virtual em Saúde, Web of Science, Scopus, PUBMED, SciELO e LITCOVID LongCovid.

**Resultados:**

Dos 13 artigos analisados, destaca-se que a assistência direcionada a pacientes com Covid longa está associada a prescrição medicamentosa, indicação de exercícios físicos, telerreabilitação e fisioterapia.

**Conclusão:**

Foi identificada uma fragmentação no cuidado prestado aos usuários dos serviços de saúde com Covid longa, com a assistência direcionada apenas a sintomas isolados, sem abordar o cuidado biopsicossocial que as pessoas com essa condição carecem.

## INTRODUÇÃO

Após a fase aguda da infecção pelo vírus SARS-CoV-2, pessoas acometidas pela doença começaram a relatar a manifestação de sintomas relacionados a complicações multissistêmicas. Esses casos foram considerados pela Organização Mundial da Saúde (OMS) como Covid longa, ocorrendo, frequentemente, três meses após o início da Covid-19 e os sintomas persistem pelo menos dois meses e não podem ser esclarecidos por um diagnóstico alternativo^(1,2)^.

Os sintomas da Covid longa são de alto grau de heterogeneidade e podem comprometer as atividades diárias e no trabalho, assim como influenciar diretamente na qualidade de vida das pessoas acometidas. Dentre os sintomas mais comuns apresentados estão a tosse persistente, febre, fadiga, dispneia, dor no peito, dores musculares, perda do olfato ou paladar, alterações no padrão de sono, comprometimento cognitivo, ansiedade e depressão^(2)^.

Existem diversas hipóteses que explicam o desenvolvimento da Covid longa, e algumas estão relacionadas à existência de reservatórios persistentes de SARS-CoV-2 em tecidos, impactos na microbiota e desregulação imunológica^(3)^. No entanto, devido à sua condição multifatorial e à diversidade de sintomas, não há um protocolo padrão para o atendimento das pessoas acometidas por essa condição, o que torna a doença um desafio para os profissionais de saúde, especialmente, no que se refere ao diagnóstico e manejo^(1,4-6)^.

Atualmente, é ofertada uma combinação de tratamentos, sendo o farmacológico direcionado ao alívio dos sintomas e os tratamentos não farmacológicos aqueles que abrangem a reabilitação para melhora das funções comprometidas, apoio psicoterapêutico e social^(3,7,8)^.

Neste contexto, a literatura tem descrito que, embora o diagnóstico da Covid longa geralmente seja realizado por um clínico geral, o manejo desses pacientes exige a cooperação multiprofissional, abordada de forma individualizada e nos âmbitos biopsicossociais, incluindo a pessoa acometida pela doença na autogestão do cuidado à saúde^(8)^.

A assistência aos pacientes com Covid longa exige uma abordagem multiprofissional com foco no cuidado integral; entretanto, por se tratar uma nova doença, ainda não se sabe como essa assistência está sendo prestada pelos profissionais de saúde, permanecendo uma lacuna no conhecimento. Cabe destacar que além do comprometimento da saúde física e mental, a Covid longa pode impactar diretamente o exercício das atividades diárias, incluindo o trabalho assim como a redução da qualidade de vida, além de representar um incremento na demanda dos serviços de saúde^(1,4-6)^.

Assim, estudos que investiguem a forma como a assistência à saúde está sendo ofertada aos pacientes com Covid longa, ou seja, dentro dos princípios do cuidado integral e centrada na pessoa ou realizada de maneira fragmentada e direcionada apenas pelos sintomas, são essenciais e inovadores visto que contribuem para identificar as potencialidades e fragilidades sobre o cenário atual da assistência às pessoas com a doença, bem como podem servir de subsídios para o fortalecimento e implementação de diretrizes para o manejo clínico dessas pessoas. Assim, este estudo tem o objetivo de mapear evidências científicas sobre a assistência à saúde ofertada aos usuários dos serviços de saúde com Covid longa.

## MÉTODO

Trata-se de uma revisão de escopo com base nos princípios delineados pelo *Joanna Briggs Institute* (JBI), que compreendem as seguintes etapas: (1) formulação da pergunta de pesquisa, (2) identificação de estudos pertinentes, (3) seleção dos estudos, (4) coleta de dados, (5) análise, resumo e relato dos resultados, e (6) divulgação dos achados^(9)^.

### Definição da Questão de Pesquisa

Para conduzir a busca na revisão, foi adotada a abordagem do JBI, representada pelo acrônimo “PCC”, que abrange os elementos de “P” (população), “C” (conceito) e “C” (contexto). A questão norteadora da pesquisa foi construída com base na estratégia PCC, sendo “P” (indivíduos com Covid longa), “C” (assistência e manejo) e “C” (serviços de saúde), definida da seguinte forma: Como está sendo direcionada a assistência aos usuários com Covid longa nos serviços de saúde?

### Estratégia de Busca e Critérios de Inclusão e Exclusão

A busca dos artigos foi realizada nas bases de dados eletrônicas: Biblioteca Virtual em Saúde, *Institute for Scientific Information* (Web of Science), *Scopus*, *US National Library of Medicine National Institutes of Health* (PUBMED), SciELO e LITCOVID LongCovid. As buscas foram realizadas entre os meses de fevereiro e março de 2023, e conduzidas por meio de descritores e seus sinônimos que constam no Descritor em Ciências da Saúde (DeCS) e no *Medical Subject Headings* (MeSH), nos diferentes idiomas, a saber: Covid longa, assistência de Enfermagem e Atenção Primária à Saúde; e, em inglês, reabilitação, Covid longa e exercício (Quadro 1).

**Quadro 1:** Estratégias de busca utilizadas nas bases de dados.

Base de Dados	Nº de Artigos	Estratégia
BIBLIOTECA VIRTUAL EM SAÚDE	59	(long covid) OR (post-covid conditions) AND (nursing care) AND (primary care) OR (primary healthcare) AND ( la:(“en” OR “pt” OR “es”)) AND (year_cluster:[2019 TO 2023])
	18	(covid de longo curso) OR (post-covid conditions) AND (nursing care) OR (cuidados de enfermería) OR (cuidados de enfermagem) AND (atenção primária à saúde) OR (primary health care) AND ( la:(“en” OR “pt” OR “es”)) AND (year_cluster:[2019 TO 2023])
WEB OF SCIENCE	643	post-acute sequelae of SARS-CoV-2 infection (Resumo) and nursing care (Resumo) and Primary Healthcare (Resumo) or Atención Básica (Resumo) or assistência de enfermagem (Resumo) or post-COVID conditions (Resumo) and Article (Tipo de documento) and English or Spanish or Portuguese (Idiomas)
	177	Long Haul COVID (Todos os campos) or post- COVID conditions (Todos os campos) and nursing care management (Todos os campos) and Cuidado Primário de Saúde (Todos os campos) or Atenção Primária à Saúde (Todos os campos) and Article (Tipo de documento) and English or Spanish or Portuguese (Idiomas)
SCOPUS	69	long AND covid OR post-covid AND conditions OR post-acute AND sequelae AND of AND sars- AND cov-2 AND infection AND nursing AND care AND management AND primary AND care OR primary AND healthcare AND ( LIMIT-TO ( PUBYEAR , 2023 ) OR LIMIT-TO ( PUBYEAR , 2022 ) OR LIMIT-TO ( PUBYEAR , 2021 ) OR LIMIT-TO ( PUBYEAR , 2020 ) ) AND ( LIMIT-TO ( DOCTYPE , “ar” ) ) AND ( LIMIT-TO ( LANGUAGE , “English” ) )
	13	long AND haul AND covid OR post-covid AND conditions AND nursing AND care AND primary AND care OR primary AND healthcare AND ( LIMIT-TO ( PUBYEAR , 2023 ) OR LIMIT-TO ( PUBYEAR , 2022 ) OR LIMIT-TO ( PUBYEAR , 2021 ) OR LIMIT-TO ( PUBYEAR , 2020 ) ) AND ( LIMIT-TO ( DOCTYPE , “ar” ) ) AND ( LIMIT-TO ( LANGUAGE , “English” ) ) AND ( LIMIT-TO ( SRCTYPE , “j” ) )
PUBMED	217	((“post acute covid 19 syndrome”[MeSH Terms] OR (“post acute”[All Fields] AND “covid 19”[All Fields] AND “syndrome”[All Fields]) OR “post acute covid 19 syndrome”[All Fields] OR (“long”[All Fields] AND “covid”[All Fields]) OR “long covid”[All Fields] OR (“post acute covid 19 syndrome”[MeSH Terms] OR (“post acute”[All Fields] AND “covid 19”[All Fields] AND “syndrome”[All Fields]) OR “post acute covid 19 syndrome”[All Fields] OR (“post”[All Fields] AND “covid”[All Fields] AND “conditions”[All Fields]) OR “post covid conditions”[All Fields]) OR (“post acute covid 19 syndrome”[MeSH Terms] OR (“post acute”[All Fields] AND “covid 19”[All Fields] AND “syndrome”[All Fields]) OR “post acute covid 19 syndrome”[All Fields] OR “post acute sequelae of sars cov 2 infection”[All Fields])) AND (“nursing care”[MeSH Terms] OR (“nursing”[All Fields] AND “care”[All Fields]) OR “nursing care”[All Fields] OR (“nursing”[All Fields] AND “care”[All Fields] AND “management”[All Fields]) OR “nursing care management”[All Fields]) AND (“primary health care”[MeSH Terms] OR (“primary”[All Fields] AND “health”[All Fields] AND “care”[All Fields]) OR “primary health care”[All Fields] OR (“primary”[All Fields] AND “healthcare”[All Fields]) OR “primary healthcare”[All Fields])) AND ((2019/12/1:2023/3/31[pdat]) AND (english[Filter] OR portuguese[Filter] OR spanish[Filter]))
	105	(((((“post acute covid 19 syndrome”[MeSH Terms] OR (“post acute”[All Fields] AND “covid 19”[All Fields] AND “syndrome”[All Fields]) OR “post acute covid 19 syndrome”[All Fields] OR (“long”[All Fields] AND “haul”[All Fields] AND “covid”[All Fields]) OR “long haul covid”[All Fields] OR (“post acute covid 19 syndrome”[MeSH Terms] OR (“post acute”[All Fields] AND “covid 19”[All Fields] AND “syndrome”[All Fields]) OR “post acute covid 19 syndrome”[All Fields] OR “post acute sequelae of sars cov 2 infection”[All Fields])) AND (“nursing”[MeSH Subheading] OR “nursing”[All Fields] OR (“nursing”[All Fields] AND “care”[All Fields]) OR “nursing care”[All Fields] OR “nursing care”[MeSH Terms] OR (“nursing”[All Fields] AND “care”[All Fields]))) OR (“atencion”[All Fields] AND (“drug effects”[MeSH Subheading] OR (“drug”[All Fields] AND “effects”[All Fields]) OR “drug effects”[All Fields] OR “de”[All Fields]) AND (“enfermeria”[Journal] OR “enfermeria”[All Fields]))) AND (“primary health care”[MeSH Terms] OR (“primary”[All Fields] AND “health”[All Fields] AND “care”[All Fields]) OR “primary health care”[All Fields] OR (“primary”[All Fields] AND “healthcare”[All Fields]) OR “primary healthcare”[All Fields])) OR ((“aten primaria”[Journal] OR (“atencion”[All Fields] AND “primaria”[All Fields]) OR “atencion primaria”[All Fields]) AND de salud[Investigator])) AND ((2019/12/1:2023/3/31[pdat]) AND (english[Filter] OR portuguese[Filter] OR spanish[Filter]))
	9	(“Rehabilitation”[All Fields] AND “long covid”[Title] AND “Exercise”[Title]) AND ((ffrft[Filter]) AND (fha[Filter]) AND (fft[Filter]) AND (english[Filter]))
SCIELO	4	(((long covid) OR (post-acute sequelae of sars- cov-2 infection) OR (post-covid conditions)) AND (nursing care)) AND (primary care) OR (primary healthcare) OR (primary health care) AND la:* AND type:(“research-article”)
	1	(post-covid conditions) OR (afecções pós-covid) OR (covid prolongado) OR (long covid) AND (assistência de enfermagem) OR (nursing care management) OR (cuidados de enfermería) AND (atenção primária à saúde) OR (primary health care) OR (primary healthcare) OR (atención básica) OR (asistencia sanitaria de primer nivel) AND la:* AND type:(“research-article”)
LITCOVID LongCovid	330	(post-acute sequelae of SARS-CoV-2 infection) AND (nursing care management) AND (Primary Healthcare OR Primary Health Care) (LIMIT-TO TITLE) AND (LIMIT-TO ENGLISH) AND (LIMIT-TO PORTUGUESE) AND (LIMIT-TO SPANISH)

### Extração e Seleção dos Estudos

Foram incluídos estudos primários, nos idiomas português, inglês e espanhol, publicados no período de dezembro de 2019 a junho de 2023, presentes nas bases de dados anteriormente citadas. Foram excluídos estudos de revisão, editoriais, protocolos, artigos de opinião, estudos cujo título e resumo não responderam à pergunta-problema, informações de *websites* e veiculadas em mídia. Também, foram examinadas as listas de referências de todos os estudos encontrados.

As referências encontradas foram importadas para o aplicativo StArt (*State of the Art through Systematic Review*) e selecionadas em dois níveis: sendo a primeira etapa de seleção a análise por meio da leitura de títulos e resumos, e posteriormente, a leitura do artigo na íntegra. A ferramenta de revisão StArt foi desenvolvida pelo Laboratório de Pesquisa em Engenharia de Software (LaPES) da Universidade Federal de São Carlos^(10)^. Os estudos elegíveis foram recuperados na íntegra e avaliados por três pesquisadores. Em ambas as etapas, as divergências foram debatidas até chegar a um consenso.

### Elaboração da Revisão

A elaboração desta revisão também aderiu às recomendações do PRISMA-ScR (*extension for scoping reviews)* para a extração dos dados^(11)^. Foram obtidos dados pertinentes de cada artigo escolhido, incluindo autores, nome do periódico, país onde o estudo foi realizado, país de publicação, desenho do estudo e principais resultados. Esses resultados são apresentados em quadros e em formato descritivo, abordando aspectos bibliométricos e respondendo à questão central que guiou esta revisão de escopo.

## RESULTADOS

Foram identificados 1645 artigos obtidos nas bases de dados. Nesta fase, o total de artigos duplicados foi de 78, sendo selecionados 1567 estudos para análise do título e resumo. A partir desta análise, foram excluídos 1501 estudos, por se tratarem de artigos de opinião, editoriais, revisões e manuais ou não responderem à questão norteadora de pesquisa. Para a leitura completa, foram incluídos 66 artigos, e por fim, foram selecionados 10 estudos para a síntese qualitativa, enquanto 3 artigos foram selecionados a partir das referências dos estudos selecionados (Figura 1).

**Figura 1:** Fluxograma de referência: inclusão e exclusão dos artigos.

A priori, foram selecionados estudos sobre assistência de enfermagem oferecida aos pacientes com Covid longa na Atenção Primária à Saúde (APS), no entanto, após o retorno de um número reduzido de estudos que abordavam o tema, ampliou-se o conceito e contexto, respectivamente, para assistência geral e serviços de saúde, sendo acrescentados 3 artigos, sendo 1 retirado, diretamente, das referências dos estudos selecionados e 2 da plataforma *US National Library of Medicine National Institutes of Health* (PUBMED).

Os estudos incluídos nesta revisão foram publicados entre 1 de dezembro de 2019 e 2 de junho de 2023, sendo que 4 (30,8%) foram realizados no Reino Unido e 1 (7,7%) em cada um desses países, a saber: Estados Unidos, Chile, em diversos países, Alemanha, Ucrânia, Espanha, Portugal, França e Áustria; e os 13 (100%) artigos foram publicados em inglês.

Dentre os 13 estudos selecionados, destacam-se 3 (23,1%), respectivamente, estudos descritivos e ensaios clínicos randomizados (Quadro 2).

**Quadro 2:** Estudos selecionados na revisão, segundo objetivo, tipo de estudo, principais resultados e estratégias e práticas utilizadas pelos estudos para realizar o manejo da Covid longa.

Autor	Objetivo	Tipo De Estudo	Principais Resultados	Estratégias e Práticas Utilizadas
Dalbosco-Salas et al.^(12)^	Analisar se o programa de telerreabilitação é eficaz no que diz respeito ao manejo da melhora da capacidade física, da qualidade de vida e de sintomas em pacientes pós-Covid adultos.	Estudo observacional prospectivo	A capacidade física, as limitações físicas, a dor corporal, as percepções gerais de saúde, a vitalidade, o funcionamento social, as limitações emocionais e a saúde mental apresentaram melhora após a intervenção, em maior parte dos pacientes. Concomitantemente, houve uma recuperação significativa da fadiga e da dispneia. Entre indivíduos que haviam sido admitidos na UTI e indivíduos que não haviam sido admitidos na UTI, todos os aspectos apresentaram melhora, com exceção de dor corporal, percepções gerais de saúde, limitações emocionais e saúde mental.	Telerreabilitação (com a oferta de exercícios físicos acompanhados por profissionais).
Daynes et al.^(13)^	Avaliar se há melhoras nas variáveis fadiga, dificuldade respiratória, capacidade de exercício físico e cognição após intervenção física, entre indivíduos com Covid longa.	Estudo observacional	O estudo indicou diminuição na capacidade de exercício e na qualidade de vida relacionada à saúde em comparação com controles saudáveis, embora a ansiedade, a depressão e a cognição estejam preservadas. Os resultados indicam uma melhora da fadiga, com o aumento do teste de caminhada incremental e de resistência, Avaliação Funcional da Escala de Fadiga para Tratamento de Doenças Crônicas, domínios EuroQual 5 (EQ5D) e Avaliação Cognitiva de Montreal e com diminuição da Escala Hospitalar de Ansiedade e Depressão.	Monitoramento clínico contínuo. No entanto, destaca-se a falta de um protocolo específico de reabilitação direcionado a pacientes com Covid longa.
Jimeno-Almazán et al.^(14)^	Comparar os resultados de pacientes com Covid longa submetidos à intervenção de exercícios terapêuticos supervisionados ou seguindo o folheto de reabilitação de autogestão da OMS (Organização Mundial da Saúde).	Estudo clínico randomizado	Foi realizada uma rotina de treinamentos simultâneos de 3 dias por semana. A partir dos sintomas prevalentes (comuns à condição, como dispneia e febre baixa), foram avaliadas as variáveis função pulmonar, composição do corpo (massa corporal gorda e magra), qualidade de vida e fadiga, ansiedade e depressão, saúde cardiovascular e força muscular. Após 8 semanas de exercício físico, evidenciou-se uma diminuição dos sinais e sintomas, principalmente no que diz respeito à dispneia. Ainda, houve uma melhora geral em todas as variáveis, no entanto, alguns resultados relatados pelos pacientes nas variáveis de fadiga e qualidade de vida não denotaram melhoras significativas entre o grupo controle, mesmo que tenha ocorrido uma melhora parcial na dispneia e funcionalidade pulmonar.	Intervenções de exercícios de aptidão física, com foco na reabilitação física supervisionada e monitorada.
Koliadenko et al.^(15)^	Estudar as manifestações clínicas de sintomas psicopatológicos em pacientes sobreviventes da Covid-19 e desenvolver um modelo conceitual para fornecer-lhes atendimento médico em telemedicina.	Estudo retrospectivo	No que diz respeito à saúde mental, nenhum dos indivíduos estudados apresentava algum indicativo de distúrbios de saúde mental ou de comportamento, anteriormente à Covid-19. Cerca de 98 pacientes (76% de todos) apresentaram comprometimento da memória. Todos os indivíduos avaliados (com exceção de um) apresentam, na época do estudo, consideráveis níveis de ansiedade, estresse e depressão, decorrentes, principalmente, da situação negativa que a pandemia da Covid-19 trouxe. Ainda, houve a identificação de sintomas hipocondríacos, decorrentes dos baixos indicadores de bem-estar, atividade e humor. O tratamento, realizado a partir da abordagem cognitivo-comportamental e associado ao uso de antidepressivos e tranquilizantes não-benzodiazepínicos, enfatizou uma melhora dos níveis de estresse, ansiedade e depressão.	Terapia Cognitivo Comportamental, sendo a assistência psicoterapêutica prestada por meio da telemedicina, tratamento medicamentoso e acompanhamento prolongado e contínuo, com monitoramento durante e após a intervenção terapêutica.
Nopp et al.^(16)^	Avaliar a eficácia e segurança da reabilitação pulmonar em ambulatório para pacientes que continuam a apresentar limitações respiratórias e/ou funcionais persistentes ou em progressão após terem contraído a Covid-19.	Estudo prospectivo	A mudança na distância de caminhada de 6 minutos aumentou (desde o início até o final da reabilitação). Após as 6 semanas do programa de reabilitação, a escala de estado funcional pós-COVID-19 diminuiu. Da mesma forma, a dispneia medida com a escala mMRC diminuiu. Ademais, os pacientes melhoraram na carga de trabalho máxima e na capacidade de resistência e a qualidade de vida aumentou. As alterações na função pulmonar e na força muscular respiratória foram exploradas após a análise inicial dos dados: no início do estudo, os pacientes apresentavam uma função pulmonar comprometida em relação à idade, sexo e valor esperado específico para a altura, com uma melhora significativa, ao final do estudo. Além disso, a pressão inspiratória máxima da boca aumentou em 28%.	Teste de caminhada de 6 minutos, conforme as diretrizes da *European Respiratory Society*.
O’Hare et al.^(17)^	Compreender como os médicos do Departamento de Assuntos de Veteranos (VA) lidaram com o diagnóstico de Covid longa e ofereceram os cuidados aos pacientes com suspeita ou confirmação de Covid longa, presentes nos registros eletrônicos de saúde (EHRs).	Estudo de coorte	Durante o manejo da Covid longa, observou-se a incerteza clínica e a fragmentação do cuidado. A incerteza clínica diz respeito à hesitação médica em diagnosticar sinais e sintomas específicos da Covid longa. Isto se dá, principalmente, por causa da multiplicidade etiológica da condição pós-Covid, da dinâmica dos tratamentos recebidos pelos pacientes, de eventos adversos à saúde que não estão relacionados à Covid-19, da deficiência da confiança médica ao estado clínico do paciente e da dificuldade do monitoramento dos pacientes afetados pela Covid longa. Quanto à fragmentação do cuidado, a assistência oferecida ao paciente é isolada e mal coordenada. Isto se dá por conta da abordagem que dá pouca importância à integralidade e às preocupações dos pacientes, da dificuldade de atuação multidisciplinar e das recomendações clínicas, que podem ser vistas como indesejáveis ou onerosas para os pacientes.	O estudo não cita as estratégias comumente utilizadas, mas destaca as práticas que favorecem a fragmentação do cuidado e as dificuldades de diagnosticar a Covid longa.
Reis et al.^(18)^	Identificar os aspectos/componentes a considerar no planejamento e na implementação de intervenções de telerreabilitação que garantam cuidados transitórios a pessoas com Covid longa, após internação e identificar os aspectos positivos da telerreabilitação.	Estudo descritivo	No estudo, as enfermeiras participantes trouxeram aspectos do manejo da Covid longa que consideram pertinentes. Em relação à coordenação entre os níveis de atenção, as participantes do estudo enfatizaram a importância de manter a continuidade da assistência, mas com cuidados transicionais, mantendo uma escuta empática, a fim de identificar as necessidades de saúde da comunidade e auxiliar os indivíduos a participarem de seu processo saúde-doença. A telerreabilitação favorece este cuidado transitório, pois, ainda, é possível assegurar todas as intervenções numa modalidade de e-saúde, no entanto, momentos presenciais são pertinentes para avaliar programas de reabilitação respiratória e motora, que devem ser realizadas com exercícios que aumentem a capacidade ventilatória, a expansão torácica, o desempenho do diafragma, o controle dos sintomas associados (tosse, dispneia e expectoração) e a tolerância ao esforço. Já a reabilitação motora deve incentivar força muscular, flexibilidade, amplitude de movimento articular, melhora da capacidade e qualidade da marcha. Ainda, a telerreabilitação mostrou-se importante no que diz respeito ao deslocamento de indivíduos, uma vez que é possível realizar o manejo das condições de saúde da própria casa, com auxílio da equipe de saúde.	Coordenação da integração entre a assistência especializada e a da Atenção Básica. Abordando a multidisciplinaridade e telerreabilitação.
Romanet et al.^(19)^	Avaliar a eficácia do treinamento físico na dispneia e na qualidade de vida relacionada à saúde em indivíduos com Covid longa.	Estudo controlado randomizado	A pontuação média do Perfil Multidimensional de Dispneia (MDP) após reabilitação do treinamento físico foi 42% menor do que após fisioterapia padrão. Ainda, reduções significativas foram observadas nas subcategorias do MDP: desconforto respiratório, dimensão sensorial e resposta emocional. Dessa forma, os resultados sugerem que a reabilitação do treinamento físico teve efeitos positivos na dispneia e na qualidade de vida, em comparação com a fisioterapia padrão em pacientes previamente hospitalizados com a síndrome do desconforto respiratório agudo devido à COVID-19.	Treinamento físico específico (caminhada e treinamentos de resistência e força muscular) e fisioterapia padrão.
Schrimpf et al.^(20)^	Avaliar o número atual de pacientes com Covid longa (pacientes com sintomas entre 4 e 12 semanas e mais de 12 semanas) tratados por clínicos gerais, bem como os sintomas observados com mais frequência em pacientes com Covid-19 aguda e Covid longa por estes médicos.	Estudo descritivo	A respeito do manejo da Covid longa, 97,2% dos profissionais de saúde abordados já trataram algum caso de sintomas persistentes entre 4 a 12 semanas da Covid-19. A capacidade de diagnosticar estes pacientes foi determinada como 62,8%, enquanto as opções terapêuticas são classificadas em torno de 47,4%. Cerca de 79,6% dos profissionais alega ter tido contato com pacientes que apresentam sintomas há mais de 12 semanas, com capacidade de diagnóstico de 40,7% e opções terapêuticas em 54,9%. Os profissionais referiram que cerca de 18,3% de todos os pacientes com Covid-19 precisa de atestado, por conta de ser incapaz de trabalhar, e, aproximadamente, 3,7% dos pacientes têm acesso a centros de reabilitação. Entre os tratamentos abordados, há opções medicamentosas, não medicamentosas (como a fisioterapia) e/ou encaminhamentos para serviços especializados.	Terapias medicamentosas e não medicamentosas, reabilitação física e encaminhamentos aos serviços especializados.
Nurek et al.^(21)^	Fornecer um guia especializado rápido para médicos e serviços clínicos de Covid longa, a partir do desenvolvimento de uma lista de recomendações.	Estudo descritivo	Entre os resultados, ressalta-se a importância do conhecimento clínico sobre a etiologia, da realização do diagnóstico a partir de critérios pré-estabelecidos, de investigações individualizadas, considerando que cada indivíduo tem as suas necessidades de saúde, de encaminhamentos adequados a centros de especialidades, de avaliações completas, com exames específicos para cada caso e de atividades de reabilitação para o manejo dos sinais e sintomas, que incluem educar o paciente sobre a sua condição de saúde, incentivando-o a participar do próprio processo saúde-doença e fazer uso de opções terapêuticas (alternativas ou não), como fisioterapia, fármacos, etc.	Abordagem multiprofissional e interprofissional, encaminhamentos a serviços especializados, considerando os aspectos fisiológicos, psicológicos e sociais da recuperação.
Ladds et al.^(22)^	Desenvolver um modelo de manejo da Covid longa, a partir de experiências de profissionais da saúde.	Estudo qualitativo em que os participantes foram convidados a escolher entre uma entrevista narrativa individual ou a participação em um grupo focal online	Foi desenvolvido, neste estudo, um modelo de sugestão para a melhoria do caráter assistencial à Covid longa, que aborda critérios de encaminhamento, encaminhamento do médico da família ou equipe de internação, triagem por telefone, com investigações que considerem os fatores sistêmicos, garantindo a integralidade, e ofereçam assistência de distintas especialidades médicas e o desenvolvimento de monitorização, acompanhamento e estímulos cognitivos.	Multidisciplinaridade, com práticas de educação continuada e integração entre serviços e continuidade do cuidado, com acompanhamento terapêutico e clínico.
Jimeno-Almazán et al.^(23)^	Determinar a eficácia do exercício físico, do treinamento muscular respiratório e do folheto de recomendações da Organização Mundial da Saúde (OMS) sobre a recuperação da aptidão física, qualidade de vida e estado dos sintomas em pessoas com a Síndrome pós-Covid-19.	Estudo de coorte, com ensaio clínico randomizado	Após 8 semanas de intervenção, que consistia em um programa de treinamento concorrente supervisionado (com treinamento muscular inspiratório ou sem ele), houve melhora significativa entre todos os participantes da pesquisa. Ocorreu uma diminuição de indivíduos que relataram ter sintomas moderados e graves, principalmente entre indivíduos que realizaram exercício físico (em comparação com os que realizaram apenas treinamento respiratório e os de controle). Houve uma baixa diminuição no grupo de controle (exceto no fator dispneia, que aumentou), mas ocorreu uma melhora considerável dos sintomas no grupo que participou da realização de exercícios físicos e uma melhora mediana nos grupos que realizaram treinamento muscular respiratório (associado ou não a exercício físico).	Prática de exercício físico.
Humphreys et al.^(24)^	Explorar a experiência vivida da Covid longa, com enfoque no papel da atividade física.	Estudo qualitativo por meio de entrevistas semiestruturadas	Para alguns participantes, a incapacitação física prolongada impactou-os emocionalmente, causando baixa autoestima, frustração e culpa por não conseguirem cumprir as atividades cotidianas. Os participantes apresentavam expectativas variadas em relação a como o sistema de saúde poderia assistenciá-los com atividades físicas, sendo que muitos sentiram-se desassistidos pelo profissional médico e procuraram informações on-line. Além disso, como atividades física ou cognitiva resultaram no aparecimento de fadiga, os participantes relataram que houve uma perda de liberdade para se envolver em atividades cotidianas. Assim, ao realizar exercícios físicos, esses indivíduos referiram recaídas, que detinham uma pequena melhora percebida na função basal, porque era considerado um preço que valia a pena pagar pelo senso de normalidade, controle e efeito positivo da atividade fornecida, por medo de efeitos adversos ou por recomendação médica. A maior parte dos participantes estabeleceu estratégias pessoais para o manejo da atividade física e muitos expressaram o desejo de um melhor monitoramento e suporte para gerenciar as atividades físicas. A maioria não conseguiu retomar atividades que antes eram centrais para sua identidade principal, então qualquer atividade que proporciona um senso de normalidade ajuda a refutar a ideia de que essa identidade alterada é permanente.	Adoção da atividade física com atendimento individualizado e monitoramento contínuo bem como suporte emocional para auxiliar nos impactos das funções diárias.

Os artigos analisados neste estudo verificaram o manejo clínico de pacientes com Covid longa, sendo que 10 (77%) estudos observaram a eficácia de exercício físico e fisioterapia para a melhora dos sinais e sintomas.

Os estudos ressaltaram a adoção, por parte dos serviços de saúde, de uma abordagem de cuidado pontual, focada nos sinais e sintomas da Covid longa e com um enfoque biomédico. Neste contexto, 6 (46,2%) estudos avaliaram o conhecimento clínico para o manejo das condições pós-Covid; e apenas 3 (23,1%) abordaram o encaminhamento a serviços especializados.

A telerreabilitação, frequentemente discutida, foi uma estratégia adotada pelos serviços de saúde para o manejo e o acompanhamento de condições biopsicossociais associadas à condição da Covid longa, por 3 (23,1%) estudos. O enfoque das demandas psicológicas foi melhor abordado em 1 (7,7%) estudo, embora estas demandas sejam citadas em outros estudos, que referem uma melhora considerável nesta variável, após as intervenções propostas. Ademais, as pesquisas selecionadas não forneceram orientações específicas para o manejo de pacientes com Covid longa, apenas analisaram ações direcionadas a um campo de atuação delimitado e não direcionaram para a análise da assistência aos sinais e sintomas de forma mais abrangente.

## DISCUSSÃO

Os estudos analisados nesta revisão enfatizam os esforços para identificar as ações da assistência à saúde direcionadas a compreender os fatores etiológicos e sanitários da Covid longa, considerando a avaliação clínica e as formas de tratamento oferecidas aos pacientes.

Os sintomas persistentes da Covid-19 apresentados com maior frequência entre os estudos avaliados são afecções respiratórias, como dispneia, dificuldade respiratória e tosse; além de comprometimento da memória, menor capacidade de lidar com frustrações, ansiedade, depressão, ataques de pânico, transtorno obsessivo-compulsivo e confusão mental. Além disso, muitos dos indivíduos apresentam fadiga, fraqueza, mialgia, capacidade física diminuída, disgeusia e anosmia^(12–20)^.

No entanto, a conduta e o manejo da Covid longa ainda são considerados imprecisos, uma vez que há uma incerteza clínica no que refere, principalmente, à identificação desses casos, que se relaciona à hesitação dos profissionais de saúde ao diagnosticar sinais e sintomas específicos associados à condição clínica. Isso impacta a conduta médica, principalmente, à multiplicidade etiológica subjacente à condição pós-Covid, à variedade de tratamentos ofertados aos usuários dos serviços de saúde, aos efeitos adversos à saúde que não são relacionados à Covid-19, à falta de confiança médica quanto ao estado clínico do paciente e às dificuldades de monitoramento dos indivíduos afetados pela Covid longa^(17)^.

Dessa forma, sendo a averiguação de possíveis diagnósticos alternativos à Covid longa limitada, nos serviços de saúde, o profissional de saúde deve considerar a ocorrência de acometimentos multissistêmicos, sendo pertinente a investigação de possíveis causas subjacentes aos sintomas desta condição clínica^(21–23)^. Portanto, destaca-se a importância de realizar uma triagem inicial atentando-se aos diversos sinais e sintomas e considerar a possibilidade de diagnóstico da Covid longa em pacientes que tiveram a confirmação clínica de Covid-19 ou o histórico de teste positivo com sintomas novos ou em evolução^(13,21)^.

Destaca-se que indivíduos com Covid longa sem diagnóstico laboratorial prévio de Covid-19 representam um desafio na prática clínica, uma vez que muitos atingidos pelo SARS-CoV-2 são assintomáticos ou não realizaram o teste RT-PCR, o que torna o diagnóstico da Covid longa mais difícil de ser realizado. Além disso, a variedade de sintomas também interfere no diagnóstico da Covid longa, uma vez que os pacientes podem apresentar desde sintomas mais comuns, como fadiga, dificuldade respiratória, tosse, desconforto nas articulações, dor no peito, dores musculares e cefaleias, até outros sintomas mais raros^(25)^.

Não obstante, devido ao prolongamento da incerteza sobre a causa dos sintomas, a evolução e o tratamento clínico dos sintomas da Covid longa, alguns profissionais de saúde adotam a realização de testes adicionais e o encaminhamento para especialistas, a fim de obter uma avaliação mais detalhada. Tais abordagens podem incluir exames de imagem ou testes diagnósticos para monitorar a recuperação do usuário do serviço e explorar possíveis causas alternativas para a sintomatologia, bem como o seguimento em serviços especializados^(17,21,22)^.

Análogo a isso, a fim de otimizar a assistência à Covid longa, é fundamental valorizar o conhecimento clínico da causa subjacente. Neste contexto, destaca-se que muitos usuários dos serviços sentem que as orientações dos profissionais de saúde são insuficientes ou mesmo não se enquadram no que desejam ouvir e buscam informações na internet, nem sempre em páginas com conteúdos confiáveis, implicando em consequências para a saúde dos mesmos. Em decorrência disso, a capacitação dos profissionais de saúde se faz essencial, especialmente no que se refere à identificação e gestão clínica da Covid longa, de forma a oferecer uma assistência integral e eficaz^(22)^.

Portanto, os serviços de saúde analisados por este estudo referem a importância do diagnóstico com base em critérios científicos e definidos, avaliações clínicas “personalizadas” para cada usuário, encaminhamentos adequados aos especialistas, investigação completa por meio de exames específicos, educação e incentivo da pessoa à participação ativa no gerenciamento da própria saúde, e do profissional de saúde, para que seja capaz de oferecer orientações confiáveis, práticas e de fácil entendimento^(21)^.

Assim, as condutas de diagnóstico da Covid longa vão de acordo com os sintomas apresentados pelo indivíduo. Dessa forma, em manifestações pulmonares deve-se seguir a avaliação clínica, testes de função pulmonar e radiografia de tórax, com a recomendação de tomografia computadorizada em casos de alterações na radiografia ou comprometimento clínico dos pulmões. Além disso, é necessário examinar o usuário e realizar exames não-invasivos (tomografia computadorizada, ecocardiografia ou ressonância magnética) para as complicações cardíacas, realizar hemogramas para as vasculares, testes de sangue e de urina e, em casos abdominais, considerar a necessidade de tomografia computadorizada ou ressonância magnética. Já em condições neuropsiquiátricas, a investigação é realizada por meio de triagem de sinais de estresse, ansiedade e depressão, e, caso necessário, por tomografia computadorizada, eletroencefalograma ou ressonância magnética^(26)^.

Apesar disso, os encaminhamentos para esses serviços especializados enfrentam desafios de coordenação e comunicação entre os níveis de assistência à saúde, especialmente em casos de sintomas mais complexos^(18,20)^. Os cuidados ofertados pelos serviços de saúde são frequentemente associados às terapêuticas anteriormente utilizadas pelos usuários, sem uma integração com outros aspectos do tratamento. Além disso, há uma delimitação de difícil compreensão dos diferentes papéis entre a equipe multidisciplinar e médicos especialistas, o que dificulta a colaboração efetiva entre eles^(17)^.

Dessa forma, por meio da análise dos estudos selecionados nesta revisão, é perceptível a fragmentação do cuidado relacionada ao manejo clínico da Covid longa, em que a assistência oferecida aos usuários é realizada de maneira isolada e com coordenação inadequada entre a articulação dos serviços sanitários. Isso ocorre devido às abordagens assistenciais que não se preocupam com a integralidade em saúde e as aflições individuais dos pacientes, e, muito menos, com a dificuldade da implementação de abordagens multidisciplinares e as orientações clínicas que podem ser indesejáveis ou onerosas para eles^(17)^.

Nesse cenário, estudo realizado em serviços da APS de Portugal mostrou que as enfermeiras buscavam garantir a continuidade do cuidado aos usuários com Covid longa por meio da coordenação entre os níveis de cuidado (assistência hospitalar e APS), incluindo visitas antes da alta hospitalar para avaliar contextos relacionados ao processo saúde-doença, bem como para preparar a família para a alta, a fim de evitar fragmentação do cuidado e interrupções no programa de reabilitação^(18)^.

Contudo, no Brasil, pelo Sistema Único de Saúde (SUS), a continuidade do cuidado e o encaminhamento ao serviço especializado enfrenta diversos desafios, como a demora nos serviços secundários e o longo tempo de espera para consultas de referenciamento, que causam ineficiência no atendimento clínico e agravamento da situação clínica do usuário destes serviços de saúde. Ainda, a baixa frequência de visitas domiciliares a pacientes incapacitados, a falta de uma relação médico-paciente sólida e a comunicação deficiente entre profissionais de saúde impactam negativamente na qualidade do cuidado prestado aos usuários da APS. Além disso, também se destacam os fatores biopsicossociais, que incluem condições incapacitantes que tornam o deslocamento difícil, dores que dificultam a locomoção, dependência de familiares para acesso às consultas, dificuldade de acesso ao transporte coletivo, baixa renda familiar e ausência de apoio financeiro e logístico da família^(27)^.

À vista disso, como uma alternativa para lidar com as necessidades em saúde que surgiram com o aparecimento da Covid longa, passou-se a evidenciar a importância da telerreabilitação. Assim, a reabilitação à distância mostrou sua relevância na mobilidade e no deslocamento dos indivíduos, permitindo o gerenciamento das condições de saúde a partir do conforto de suas casas e com o suporte da equipe de saúde. Desse modo, para pessoas que enfrentam dificuldades de mobilidade, a acessibilidade a cuidados especializados pode ser de difícil gerenciamento e a telerreabilitação emerge como uma solução, utilizando tecnologias de comunicação para oferecer suporte contínuo e reabilitação a pessoas em áreas geograficamente distantes. Ressalta-se que este método não visa substituir as avaliações tradicionais, mas proporcionar uma abordagem alternativa para a assistência em saúde, sendo que já era utilizado no período pré-pandêmico, mas foi amplamente difundido durante e após a fase crítica da Covid-19^(12,18,22,28)^.

Em relação às vantagens, a telerreabilitação domiciliar é uma opção adotada pelos serviços de saúde, quando usada para monitorar ou avaliar usuários destes serviços durante a terapia corretiva, o que é benéfico para indivíduos com condições graves, proporcionando a realização da fisioterapia em casa e evitando o deslocamento até uma unidade de saúde. Apesar disso, a desigualdade no acesso a essa tecnologia é um problema significativo, especialmente em países de baixa e média renda, além de poder ocorrer, também, a falta de contato humano direto com o médico, uma vez que a interação clínica é realizada por meio de tecnologia. Além disso, para cada usuário, os profissionais de saúde precisam adaptar a intervenção à distância para atender às necessidades individuais de tratamento^(29,30)^.

Anteriormente à pandemia de Covid-19, um estudo realizado nos Estados Unidos, com indivíduos em uso de cadeira de rodas, evidenciou que um programa de telerreabilitação atenuou obstáculos associados aos cuidados convencionais, como o deslocamento de suas próprias casas e o acesso a especialistas. Por meio de sessões interativas e avaliações em tempo real, o programa ofereceu uma educação de autogestão eficaz. Ademais, a observação dos participantes em seus ambientes domiciliares favoreceu a identificação e o manejo de prováveis obstáculos que, potencialmente, poderiam afetar o seu tratamento, resultando em abordagens mais rápidas^(31)^.

Igualmente, as abordagens de telerreabilitação são tão eficazes quanto as presenciais, principalmente em programas de gerenciamento de condições crônicas^(32)^. A telerreabilitação favorece os cuidados transitórios, uma vez que é possível manter as intervenções assistenciais em uma modalidade de *e-saúde*, embora ocorram momentos em que a interação presencial é necessária, principalmente para avaliar e realizar programas de reabilitação respiratória e motora^(18)^.

Assim, a telerreabilitação melhora o acesso aos programas de reabilitação, oferecendo maior conforto aos pacientes com Covid longa e proporcionando a continuidade do cuidado. Cabe ressaltar, que a fase crítica da pandemia impulsionou a adoção das soluções remotas de saúde, permitindo que os profissionais de saúde se adaptassem à nova realidade e oferecessem um suporte mais abrangente e acessível aos usuários dos serviços de saúde, incluindo os programas de reabilitação de acordo com as necessidades individuais. Ainda, no contexto da Covid longa, a telerreabilitação incentiva uma maior autonomia e controle do próprio processo saúde-doença, incluindo estratégias de autogestão. Nessa perspectiva, a inserção de um programa de telerreabilitação, incluindo a autogestão pelos serviços de saúde, pode proporcionar uma melhora na capacidade física, saúde geral, vitalidade, função social e saúde mental em parte dos pacientes acometidos com a Covid longa, bem como a melhoria da fadiga e da dispneia^(12,18)^.

Por outro lado, um estudo enfatizou a necessidade de atendimentos presenciais de pessoas com Covid longa, devido a exigência de monitoramento contínuo, assistência integral e análise de sinais e sintomas, bem como pela dificuldade de realização da avaliação clínica e comunicação, ocasionada pela modalidade remota^(22)^.

Concomitantemente, reabilitações de exercícios físicos moderados são frequentemente indicadas, com a finalidade de aliviar os sintomas e melhorar o desempenho físico (embora seja necessário considerar as limitações e possíveis recidivas de manifestações clínicas da Covid longa, exigindo planos de ações individualizados, a fim de evitar a volta de sinais e sintomas). Nesse sentido, a abordagem multidisciplinar de reabilitação é a ideal, contemplando uma perspectiva holística que abrange as complexas sequelas da infecção por SARS-CoV-2^(20)^.

Estudos sobre a reabilitação da Covid longa por meio da realização de exercícios físicos também evidenciaram redução de sentimentos negativos, como a ansiedade e a depressão, melhoras significativas na qualidade de vida, retorno às atividades diárias e redução de sintomas como tosse, fadiga, comprometimento de memória e dispneia. Destaca-se que exercícios de alta intensidade (isto é, sessões mais longas com exercícios-alvo de maior intensidade) são considerados o tipo de tratamento de reabilitação mais apropriado para indivíduos com falta de ar após a Covid-19, uma vez que a qualidade de vida relacionada à saúde fisiológica apresenta melhora, quando associada a esta variável^(13,19)^.

Em uma intervenção de 8 semanas em um serviço ambulatorial, foi verificada uma redução no número de pessoas que relataram sintomas moderados e graves da Covid longa, especialmente entre aqueles que praticaram exercícios físicos^(23)^. Ainda, as condições funcionais dos pacientes pós-Covid do serviço em reabilitação física melhoraram, com diminuição de sintomas prevalentes da condição como a dispneia, a fadiga, a ansiedade e a depressão, aumento da capacidade de resistência, da composição corporal e na qualidade de vida, e, também, houve uma recuperação significativa na função pulmonar e na força muscular respiratória^(14,16)^.

A fadiga resultante de atividades físicas ou cognitivas pode limitar a participação em tarefas cotidianas, levando a uma sensação de perda de liberdade. Assim, apesar de recaídas com exercícios físicos, a maioria considera o esforço físico compensatório, pelo senso de normalidade e controle, buscando atividades para contrabalançar os efeitos adversos^(24)^.

Nesse contexto, associados à reabilitação física, há a adoção do uso de medicamentos como uma estratégia de manejo da Covid longa, como os analgésicos não opioides (paracetamol e o ibuprofeno) para o alívio de dores de cabeça, além do emprego de corticoides ou broncodilatadores, a fim de tratar alguns sintomas respiratórios^(20)^. Além disso, há considerações sobre o uso de antidepressivos para aliviar os sintomas persistentes de caráter inflamatório, com foco na redução de marcadores inflamatórios e na restauração da função imunológica^(33)^. Outrossim, com o propósito de reduzir a persistência de sinais e sintomas flogísticos, pacientes com Doença Pulmonar Intersticial (DPI) ocasionada pela Covid longa têm utilizado prednisona, que leva a uma melhora rápida e significativa na falta de ar e na função pulmonar^(34)^.

Recomendam-se, também, suplementos vitamínicos, principalmente a vitamina D e vitaminas do complexo B^(20)^. Portanto, é fundamental realizar uma avaliação completa por meio da anamnese, do histórico médico e de exames, a fim de obter uma avaliação abrangente, incluindo análises de sangue, função renal, proteína C-reativa, função hepática, tireoide, hemoglobina A1c (HbA1c), vitaminas D e B12, magnésio, folato e níveis de ferritina^(33)^.

No contexto brasileiro, evidencia-se a importância da APS no que diz respeito ao manejo da Covid longa, uma vez que indivíduos que apresentam sintomas persistentes da Covid-19 tendem a buscar atendimento nesse nível de assistência para avaliação clínica, realização de exames e, se for o caso, indicação de reabilitação por outras especialidades, assim como o encaminhamento para a atenção especializada ou hospitalar^(35)^.

Apesar disso, verifica-se uma minoria de pacientes conseguindo acessar os serviços de reabilitação em saúde. Assim, é preciso garantir o acesso às ações de intervenção, tangendo aos princípios do SUS, e, também, mantendo a continuidade do cuidado. Outro desafio relaciona-se à falta de conhecimento sobre a Covid longa, que pode favorecer a baixa adesão aos serviços de reabilitação, visto que muitos usuários dos serviços podem negligenciar os sintomas. Outrossim, evidencia-se uma falta de preparo e organização dos serviços de saúde brasileiros durante a fase de emergência sanitária imposta pela Covid-19, bem como verifica-se uma falta de conhecimento e padronização da assistência às pessoas que apresentam sintomas característicos da Covid longa^(36)^.

Além disso, é necessário manter um atendimento interdisciplinar, com a finalidade de realizar não apenas a reabilitação física, mas a mental e a social^(37)^. A reabilitação psíquica de sintomas persistentes da Covid-19 nos serviços de saúde que a adotam refere-se a um tratamento baseado na abordagem cognitivo-comportamental e complementado por antidepressivos e tranquilizantes não-benzodiazepínicos, que enfatiza uma melhora dos níveis de estresse, ansiedade e depressão, apesar de, em situações de complicações cardiovasculares, excretoras ou outras manifestações sistêmicas durante a fase aguda da Covid-19, ocorrer maior complexidade cognitiva, demandando suporte psicológico mais profundo^(15)^. Dessa forma, a perspectiva biopsicossocial da saúde é a estratégia mais eficaz para fornecer a assistência em toda a sua integralidade, considerando os fatores psicológicos e sociais do usuário dos serviços, não apenas os aspectos biomédicos^(38)^.

Apesar disso, o exercício físico, principalmente o aeróbico, adotado como estratégia pelos serviços de saúde, apresenta melhorias substanciais na saúde mental de indivíduos com Covid longa, em comparação com os submetidos a tratamentos tradicionais ou que não praticam atividades. Isto ocorre porque a prática de exercícios pode desencadear uma série de alterações interligadas no encéfalo, resultando em um ambiente que oferece proteção contra a depressão e a ansiedade^(39)^.

Portanto, torna-se essencial assegurar o acesso aos serviços que promovem a saúde mental para os pacientes com Covid longa, sendo necessário identificar aqueles que requerem suporte adicional, encaminhando-os ao tratamento especializado. Contanto, é importante evitar a patologização do indivíduo, considerando que os sintomas físicos da Covid-19 podem influenciar na avaliação^(40)^. Dessa forma, apesar de haver protocolos para estratégias isoladas, como reabilitação física, faz-se necessário o desenvolvimento e a implementação de diretrizes específicas de manejo que considerem a integralidade em saúde de pacientes acometidos pela Covid longa^(13)^.

Nesta pesquisa, os resultados evidenciam a reverberação a longo prazo da pandemia da Covid-19 e o impacto nos serviços de saúde, exigindo a formulação de diretivas relacionadas ao cuidado e ao tratamento da Covid longa, além da necessidade do direcionamento de recursos e esforços para abordagens mais eficazes, visto que há uma evidente lacuna na literatura sobre a forma de identificação e manejo clínico das pessoas com essa condição.

## CONCLUSÃO

Essa revisão de escopo permitiu identificar que a assistência aos pacientes com Covid longa nos serviços de saúde tem sido direcionada significativamente aos sinais e sintomas da doença, permeada pelo modelo biomédico, evidenciando assim, a ausência do cuidado integral de que a pessoa que desenvolve a doença necessita, visto que os impactos da doença vão muito além da saúde física.

Portanto, este estudo contribui para fortalecer o arcabouço teórico sobre a fragilidade no que tange uma assistência integral direcionada aos pacientes com Covid longa nos serviços de saúde bem como para a elaboração e implementação de protocolos de manejo clínico da doença que orientem à importância de uma coordenação multiprofissional no cuidado integral dessas pessoas. Por fim, sugere-se que estudos futuros trabalhem na identificação das dificuldades dos profissionais de saúde para oferecer um cuidado integral aos usuários dos serviços de saúde com Covid longa.
